# Editorial: Sustainable catalytic production of bio-based heteroatom-containing compounds — volume II

**DOI:** 10.3389/fchem.2022.1008895

**Published:** 2022-09-05

**Authors:** Jinshu Huang, Hu Li, Yaqiong Su, Song Yang

**Affiliations:** ^1^ State Key Laboratory Breeding Base of Green Pesticide and Agricultural Bioengineering, Key Laboratory of Green Pesticide and Agricultural Bioengineering, Ministry of Education, State-Local Joint Laboratory for Comprehensive Utilization of Biomass, Center for R&D of Fine Chemicals, Guizhou University, Guiyang, China; ^2^ School of Chemistry, Xi’an Key Laboratory of Sustainable Energy Materials Chemistry, State Key Laboratory of Electrical Insulation and Power Equipment, Xi’an Jiaotong University, Xi’an, China

**Keywords:** sustainable chemistry, biomass conversion, catalytic mechanism, biorefinery, biofuels

Fossil-based heteroatom-containing compounds are crucial core scaffolds or key intermediates in a wide range of pharmaceutical molecules, fiber dyes and printing ink (Li et al., 2019a; Wu et al., 2020; Wu et al., 2021), which can also be directly used as solvents, surfactants, and so on (Li et al., 2019b; Wu et al., 2019; Xu et al., 2019). However, mainly owing to the dependence and overuse of fossil source, the environmental pollution/deterioration and greenhouse effect are becoming increasingly prominent. In this regard, sustainability is deemed as a key parameter for the future of producing heteroatom-containing compounds and environmental enhancement, which not only requires the replacement of the fossil fuel feedstocks by other renewable resources (e.g., non-food biomass), but also needs the adoption of circular utilisation methods to prevent leakage of any ingredient into the environment (Iglesias et al., 2020).

This Research Topic is Volume II of a series, and here we present a collection of original research and review articles (20 papers in total) with topics on green and sustainable chemistry, including catalytic conversion of biomass feedstocks (Liu et al., Zhu et al., Liu et al., Zhang et al., Sun et al., Yao et al., Zhou et al., Zhao et al., Yang et al., and Zhou et al.), biodiesel production (Wu et al., Zhang et al., and Liu et al.), and green synthesis of heteroatom-containing bioactive compounds and functional materials (Wang et al., Pan et al., Chen et al., Pan et al., Zhang et al., and Bao et al.). Also, the Research Topic provides interesting insights into the green photocatalysis of organic pollutants (Zhang et al.).

Original research paper of Zhou et al. reports the controllable preparation of three kinds of Fe-based composite catalysts with different Fe loadings for efficient hydrogen production from biomass steam gasification. When the mass fraction of loading is 10%, Fe species are well dispersed on the carrier, affording a high gas yield of 60.4% (with 42.2% hydrogen proportion). Zhang et al. use glycerol waste to comparatively evaluate the ameliorative effect on lignocellulose under microwave or conventional heating method. During fast pyrolysis, levoglucosan produced from microwave-treated samples (32.9%) was far more selective than the conventional heating group (18.8%), and the content of aldehydes (high toxicity to the downstream fermentation) after glycerol waste and microwave pretreatment was decreased by 2.5 times compared with the untreated counterpart. In addition to directly using raw biomass resources, simple sugars like fructose can be efficiently converted to 5-hydroxymethylfurfural (up to 82% yield) by dehydration over a stable Ti-doped SBA-15 catalyst in DMSO at 140°C for 1 h (Zhu et al.), or to 5-ethoxymethylfurfural (80.4% yield) by cascade dehydration-etherification using a UIO-66-SO_3_H catalyst in ethanol under the same thermal conditions (Zhao et al.). Zhang et al. manufacture a biomass-based solid acid catalyst (SiO_2_@Cs-SO_3_H) with a large specific surface area (21.82 m^2^/g) and acidity (3.47 mmol/g) using renewable chitosan as raw material through sulfonation procedure under relatively mild conditions, which is active for esterification of oleic acid and methanol to produce biodiesel (98.2% yield).

The work of Chen et al. reports an unprecedented inactivation process of the indanol-derived NHC catalysts bearing N-C_6_F_5_ groups, giving an unexpected multi-cyclic complex product from the 3-component reaction with 1-methylcyclopropyl-carbaldehyde, 2,2,2-trifluoroacetophenone and the NHC catalyst. Pan et al. develop an acid-catalyzed 2-alkylation of indole molecules catalyzed by traceless HI, and 2,3-disubstituted indole molecules bearing congested tertiary carbon centers are obtained in moderate to good yields. Some functional catalytic materials such as hierarchical porous SAPO-34 (Wang et al.), bimetallic Zn-Zr metal-organic framework (Zhang et al.), and graphene oxide-silver nanoparticles composite (Bao et al.) are prepared in sustainable ways, and found to be efficient for the synthesis of value-added chemicals (e.g., 4,6-dimethyldibenzothiophene) or degradation of organic pollutants.

This Research Topic features several review articles with distinct scopes (Liu et al., Sun et al., Zhou et al., Yang et al., Yao et al., Liu et al., Wu et al., Liu et al., Pan et al., and Zhang et al.). Liu et al. review the application of recyclable heterogeneous non-noble Zr/Hf-containing catalysts (e.g., Zr/Hf-containing metal oxides, supported materials, zeolites, metal organic frameworks, metal organic hybrids) with acid-base bifunctionality for catalytic transfer hydrogenation using the safe liquid hydrogen donor, with emphasis on evaluating the reaction mechanisms and conversion pathways. In a more detailed manner, the research progress of catalytic synthesis of *γ*-valerolactone from furfural by Zr/Hf-based catalysts is reviewed by Sun et al., and the effects and regulation approaches of Lewis acid-base and Brønsted acid sites in the catalysts on each steps in the reaction process are discussed. Zhou et al. reveal the significance and potential of using titanate nanotubes-based materials as sustainable and environmentally benign solid catalysts/supports for synthesis of various bio-based chemicals, such as glycerol-derived solketal, jet fuel range alkanes precursors, biomass-derived esters, aldehydes, and aromatic compounds. Yang et al. propose the research development trend for improving the institutional mechanism of the utilization of crop straw resources, strengthening technology research and development, exploring the economic model of green cycle agriculture, accelerating the construction of the industrial system, and designing new paths of resource utilization in multiple ways. Yao et al. mainly review some latest studies about the conversion of cellulose to 5-hydroxymethylfurfural catalyzed by solid acids with Brønsted and/or Lewis acidic sites, such as sulfonated solid acids, carbon-based acids, and zeolites. Liu et al. summarize the mechanisms of several important processes of producing 5-ethoxymethylfurfural from lignocellulosic biomass-derived sugars and the research progress of the developed acid catalysts. In addition, advancements in tobacco (*Nicotiana tabacum L.*) seed oils (Wu et al.) and lipid extraction from microalgae using green solvents (Liu et al.) for biodiesel production are also collected. For some structurally somplex natural products such as sex pheromones (Pan et al.), and momilactones and related 9β-H pimarane skeleton (Zhang et al.), the recent advances in their synthetic strategies with the involved challenges are overviewed.

We wish this Research Topic attracts interested colleagues, enlightening more eco-friendly and sustainable synthetic procedures, shedding light on renewed catalytic strategies and routes developed for the production of bio-based heteroatom-containing compounds, and providing enthusiasm in research and studies. Enjoy its reading!
